# I-125 seeds brachytherapy with transcatheter arterial chemoembolization for subcapsular hepatocellular carcinoma

**DOI:** 10.1186/s12876-022-02356-0

**Published:** 2022-06-01

**Authors:** Fu-Lei Gao, Yong Wang, Xiang-Zhong Huang, Tian-Fan Pan, Jin-He Guo

**Affiliations:** 1 Department of Interventional Radiology, Affiliated Jiangyin Hospital, Medical College of Southeast University, Jiangyin, China; 2 Center of Interventional Radiology and Vascular Surgery, Department of Radiology, Zhongda Hospital, Medical School, Southeast University, Nanjing, China

**Keywords:** Hepatocellular carcinoma, Subcapsular, I-125 seed, Transarterial chemoembolization

## Abstract

**Background:**

I-125 seeds brachytherapy (ISB) has been used to improve the clinical effectiveness of transarterial chemoembolization (TACE) for hepatocellular carcinoma (HCC). We aim to appraise the safety and clinical efficacy of combined ISB and TACE for the treatment of subcapsular HCC.

**Materials and methods:**

A retrospective investigative study extending from January 2017 to December 2020, involved individuals suffering from subcapsular HCC, who were subjected to TACE treatment with or without ISB in our center. The clinical effectiveness was compared between 2 groups.

**Results:**

Sixty-four patients, in total, with subcapsular HCC had to undergo TACE with (n = 32) or without (n = 32) ISB in our center. After CT-guided ISB, only 2 (6.3%) patients experienced a self-limited pneumothorax. Combined treatment resulted in a significantly higher complete response (56.3% vs. 18.8%, *P* = 0.002) and total response (90.7% vs. 59.4%, *P* = 0.004) rates than that of TACE alone. In comparison to the TACE alone group, the median progression-free survival was substantially longer in the combined treatment group (11 months vs. 5 months, *P* = 0.016). Further, 15 and 28 patients in combined and TACE alone groups respectively died within the follow-up. The median OS was comparable between combined and TACE alone groups (22 months vs. 18 months, *P* = 0.529).

**Conclusions:**

Combined TACE and ISB therapy is a safe treatment method for individuals suffering from subcapsular HCC. When compared, combined treatment had significantly enhanced clinical efficacy as a subcapsular HCC therapy, in comparison to TACE alone.

## Background

**A**pproximately 90% of primary liver cancers comprise hepatocellular carcinoma (HCC) and are evidently a major health issue around the world [1–3]. Despite the fact that surgical resection is the optimal treating pathway for HCC, in the majority of cases (> 60%), diagnosis takes place at the developed stage of the tumor when surgery is no longer suggested [4–6].

For advanced-stage HCC patients, transarterial chemoembolization (TACE) is an efficacious therapeutic strategy, with a 1-year overall survival (OS) rate of 52.6–57.5% [7–10]. Several treatments which include percutaneous ablation, radiotherapy, or systemic therapy (Sorafenib or immunotherapy) have the potential to improve the clinical effectiveness of TACE alone, and the 1-year OS rate of combined treatments can reach up to a maximum of 71.9–77.5% [7–10].

Percutaneous ablation is recommended currently for small HCCs and is considered as an adequate alternative treatment to surgery [11]. However, the utilization of percutaneous ablation in subcapsular HCC is usually rendered unsafe on account of its proximity to the diaphragm and bowel and adjacent viscera [12]. Therefore, some researchers used I-125 seeds brachytherapy (ISB) instead of percutaneous ablation for the subcapsular HCC [13]. At present, the number of investigations, concerning the utilization of TACE with ISB for subcapsular HCC is still limited.

The main objective of the current works is the evaluation of the clinical safety and effectiveness of combined TACE and ISB for subcapsular HCC.

## Methods

### Patients selection

**Fig. 1:**
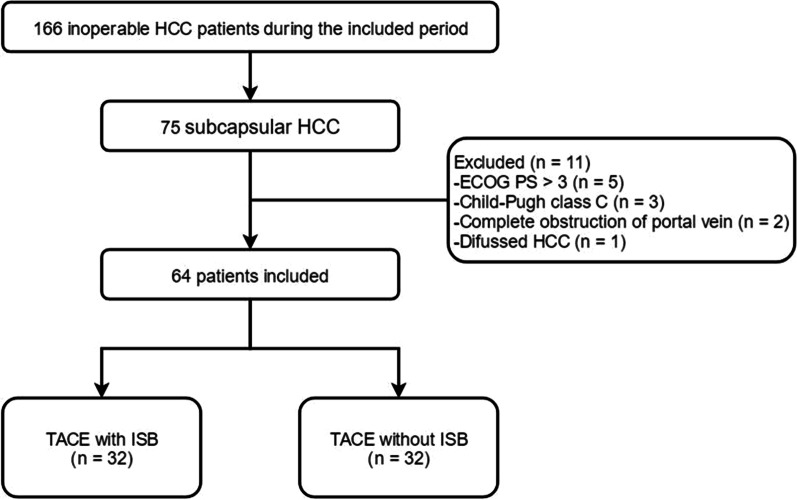
The flowchart of this study

This survey is a retrospective investigation from a single-center and it was confirmed through our Institutional Review Board. However, informed satisfaction from the patient was waived. From January 2017 to December 2020, patients suffering from subcapsular HCC were given TACE with or without ISB in the center (Fig. 1). The inclusion criteria comprised: (a) a diagnosis of subcapsular HCC; (b) inoperable cases or the patients who refused the surgical treatment; (c) number of tumors in individual patient ≤ 3; (d) Eastern Cooperative Oncology Group (ECOG) performance status (PS) 0–2; and (e) Child–Pugh liver function class A or B. The exclusion criteria comprised: (a) patients who had undergone liver surgery, chemoradiotherapy, TACE, or ablation previously; (b) diffused HCC; (c) for the individuals having multiple HCCs if the subcapsular HCC was not the dominant tumor, they were excluded; (d) complete obstruction of the portal vein; and (e) life expectancy < 3 months.

Diagnosis.

Confirmation of HCC diagnosis was on the basis of Management of hepatocellular carcinoma: an update [14]. The diagnosis of HCC was confirmed by dynamic contrast-enhanced computed tomography and/or magnetic resonance imaging based on the typical enhancement pattern (arterial hypervascularity and venous/delayed phase washout). Biopsy was performed if the imaging findings were equivocal. Accordingly, When the distance between tumor margin and the liver surface was < 10 mm, it was referred to as subcapsular HCC [12]. Subcapsular HCCs can be divided into exophytic and non-exophytic. The exophytic tumor was defined as the tumor protrusion beyond the liver surface [12].

### TACE treatment

Using local anesthesia, the TACE procedures were carried out under fluoroscopic guidance. The right femoral artery was punctured. The tumor blood supply arteries were confirmed via angiography using a 5F catheter (Terumo, Tokyo, Japan). A roadmap was established based on the intraoperative angiography. Then, the 2.7F micro-catheter (Terumo) was inserted via the 5F catheter and placed into the segmental or subsegmental hepatic arteries supplying blood to the tumors under the guidance of the roadmap. TACE was performed with the mixture of 5-fluorouracil (150 mg), mitomycin C (10 mg), epirubicin (50 mg), and lipiodol (10–20 ml). A gelatin sponge was employed initially to embolize the arteriovenous fistula, in cases where it was present. After TACE, angiography was carried out again to confirm whether there was residual tumor staining.

### Treatment planning of ISB

ISB was usually performed 2 weeks after TACE. Each I-125 seed (4.5-mm long, 0.8-mm diameter) emitted a 35.5-keV low-energy γ-rays, having a half-life equivalent to 59.6-day, an activity of 0.6–0.8 mCi (Chinese Atomic Energy Science Institution, Beijing, China), and an incipient rate of 7 cGy/h.

**Fig. 2:**
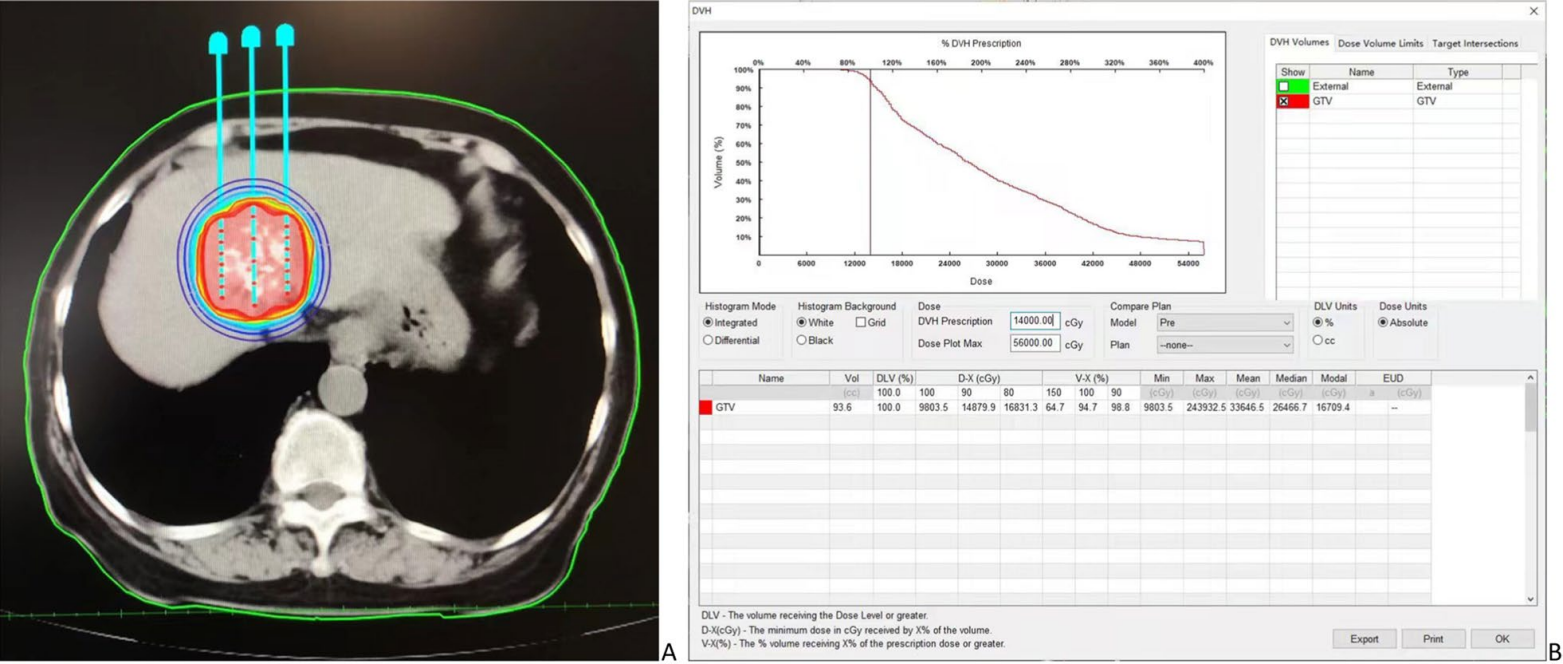
The **a** isodose curves plotted by the TPS and **b** dose-volume histogram

A 16-row CT scan (Philips, Cleveland, Ohio, USA) was used to assess HCC prior to ISB. The CT imaging information was sent to a treatment-planning system (TPS; BT-RSI; YuanBo, Beijing, China). Tumors were contoured manually on all axial CT slices and the gross tumor volume (GTV) was assessed by the TPS system. The prescribed dose was 100–140 Gy. The TPS automatically evaluated the estimated number of I-125 seeds and optimized the related spatial distribution. The planning goal was that 90% of the GTV could achieve the prescribed dose. The curves of isodose and histogram of dose-volume were shown in Fig. 2.

### CT-guided ISB

**Fig. 3:**
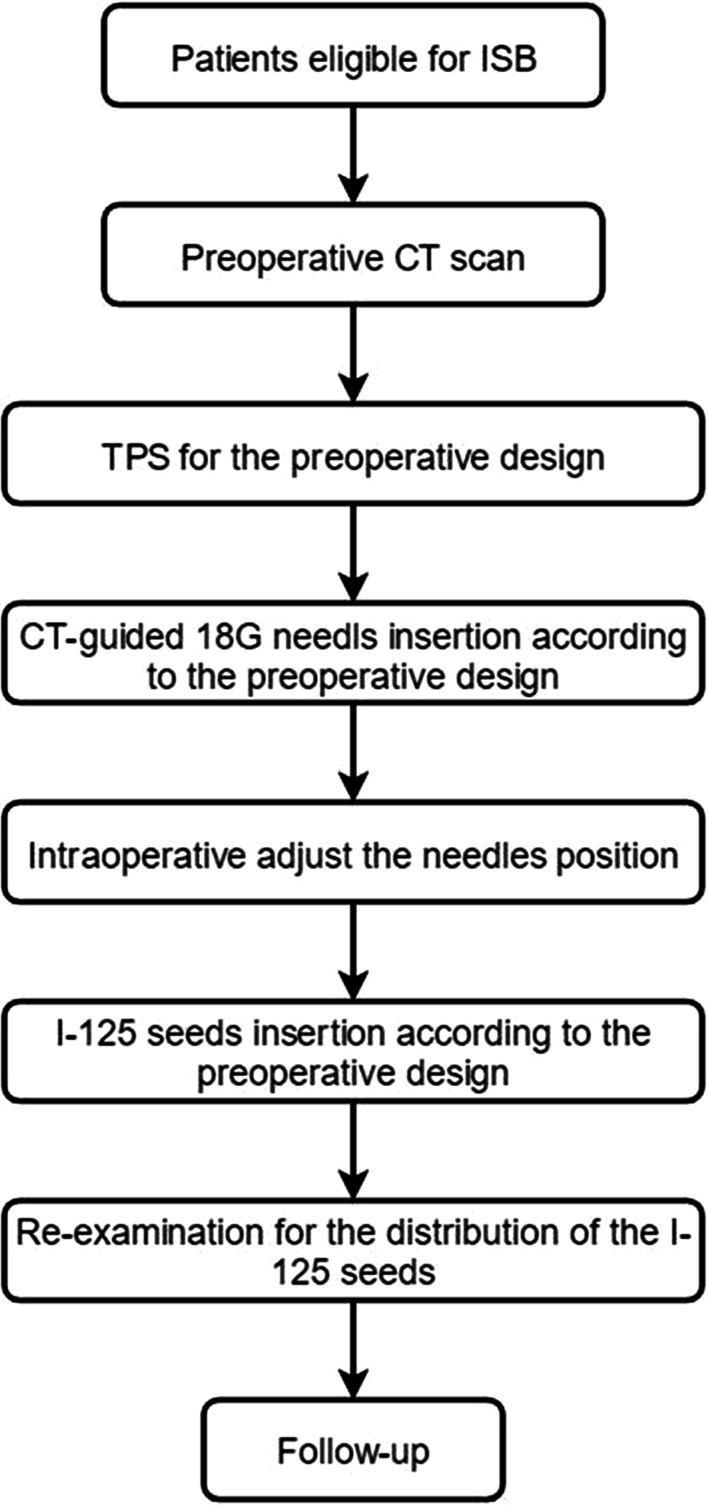
The flowchart of the CT-guided ISB procedures

The ISB was carried out under CT guidance with local anesthesia. The tumor location determined the position of the patients. One or multiple 18G needles were used to insert the I-125 seeds. The needle pathways were designed by the TPS system. When the needles were embedded into the tumor, the I-125 seeds were placed into the tumor according to the treatment plan. The I-125 seeds were implanted one after another, with a 5–10 mm space between seeds. The needles were withdrawn so that the I-125 seeds were dropped along the needle pathways. The distribution of the seeds was confirmed by an additional CT scan. The flowchart of the CT-guided ISB procedures was shown in Fig. 3.

### Follow-up

The follow-up was conducted at 1, 3, and every 3 months after treatment. The follow-up was concluded at death or till a point of time in December 2021. The investigations included in the follow-up were magnetic resonance imaging (MRI) or abdominal contrast-enhanced CT, routine blood examination, liver function test, and tumor marker test (AFP, CEA, CA125, and CA199). Repeat TACE was performed, if there was CT/MRI enhanced area in the treated tumor.

### Treatment response

Treatment response was used to assess the short-term effectiveness [4], and it was appraised in compliance with the altered response assessment criteria in solid tumors (mRECIST) [15].

Complete response (CR): the disappearance of any intratumoral arterial enhancement in all target lesions.

Partial response (PR): Minimum of 30% decrease in the sum of diameters of viable (improvement in the arterial phase) target lesions, considering the baseline sum of the diameters of target lesions as reference.

Stable disease (SD): any cases that do not qualify for either progressive disease or partial response.

Progressive disease (PD): an improvement of the minimum of 20% in the sum of the diameters of viable (enhancing) target lesions, considering the smallest sum of the diameters of viable (improving) target lesions recorded since the starting point of treating procedure, as reference.

The time from the first TACE process until death from any cause or the last follow-up was referred to as overall survival (OS). The absence of any new intrahepatic or extrahepatic lesions, local progression, or death was classified as progression-free survival (PFS) [16].

### Statistical assessment

SPSS v16.0 (SPSS, Inc., Chicago, Illinois, USA) was used for statistical analyses. The χ^2^ test or Fisher exact test were employed for the evaluation of the categorical variables and the t-test was employed for the evaluation of the continuous variables. To compare the rates of OS and PFS between groups, the curves of Kaplan–Meier and the test of log-rank were made use of. A multivariate Cox regression assessment was employed for identifying variables linked to OS and PFS, with all variables with a *P* < 0.1 in initial univariate analyses being included in the final multivariate model. *P* < 0.05 was set as the level of statistical significance.

## Results

### Patients

A total of 64 patients with subcapsular HCC underwent TACE with (n = 32) or without (n = 32) ISB in our center (Table 1). All HCCs were diagnosed based on the typical imaging findings. Therefore, biopsy was not required. Except for the rate of HBsAg (+), the other baseline parameters were comparable among the two groups. All patients with HBsAg (+) received antiviral therapy. Furthermore, 4 cases in the combined group had extra-hepatic metastasis, which included lung metastasis (n = 3) and adrenal metastasis (n = 1). Four and 3 cases in combined and TACE alone groups had PVTT, respectively, and all of the 7 cases had the branched PVTT. During the follow-up, the mean cycles of TACE were 2.8 and 5.0 in the combined and TACE alone groups, accordingly (*P* = 0.001).

**Table 1:** Patient characteristics in 2 groups

	Combined treatment	TACE alone	*P* value
Patients number	32	32	–
Age (years)	62.7 ± 11.8	62.1 ± 13.3	0.895
Gender			1.000
Male	26	26	
Female	6	6	
HBsAg (+)	22	31	0.003
AFP (mg/ml)			0.281
≥ 400	24	20	
< 400	8	12	
Diameter (cm)	5.5 ± 1.9	5.8 ± 2.7	0.627
Number of tumor			0.146
Single	25	19	
Multiple	7	12	
ECOG PS			0.091
0	23	16	
1	7	11	
2	1	5	
BCLC stage			0.737
A	16	14	
B	12	15	
C	4	3	
Child–Pugh class			0.756
A	26	25	
B	6	7	
Exophytic cases	11	7	0.266
PVTT	4	3	1.000
Extra-hepatic meatastasis	4	0	0.121
Target treatment			0.412
Yes	8	11	
No	24	21	

*ECOG PS* Eastern Cooperative Oncology Group performance status, *AFP* Alphafetoprotein, *BCLC* Barcelona clinic liver cancer, *PVTT* portal vein tumor thrombi, *TACE* transcatheter arterial chemoembolization

**Fig. 4:**
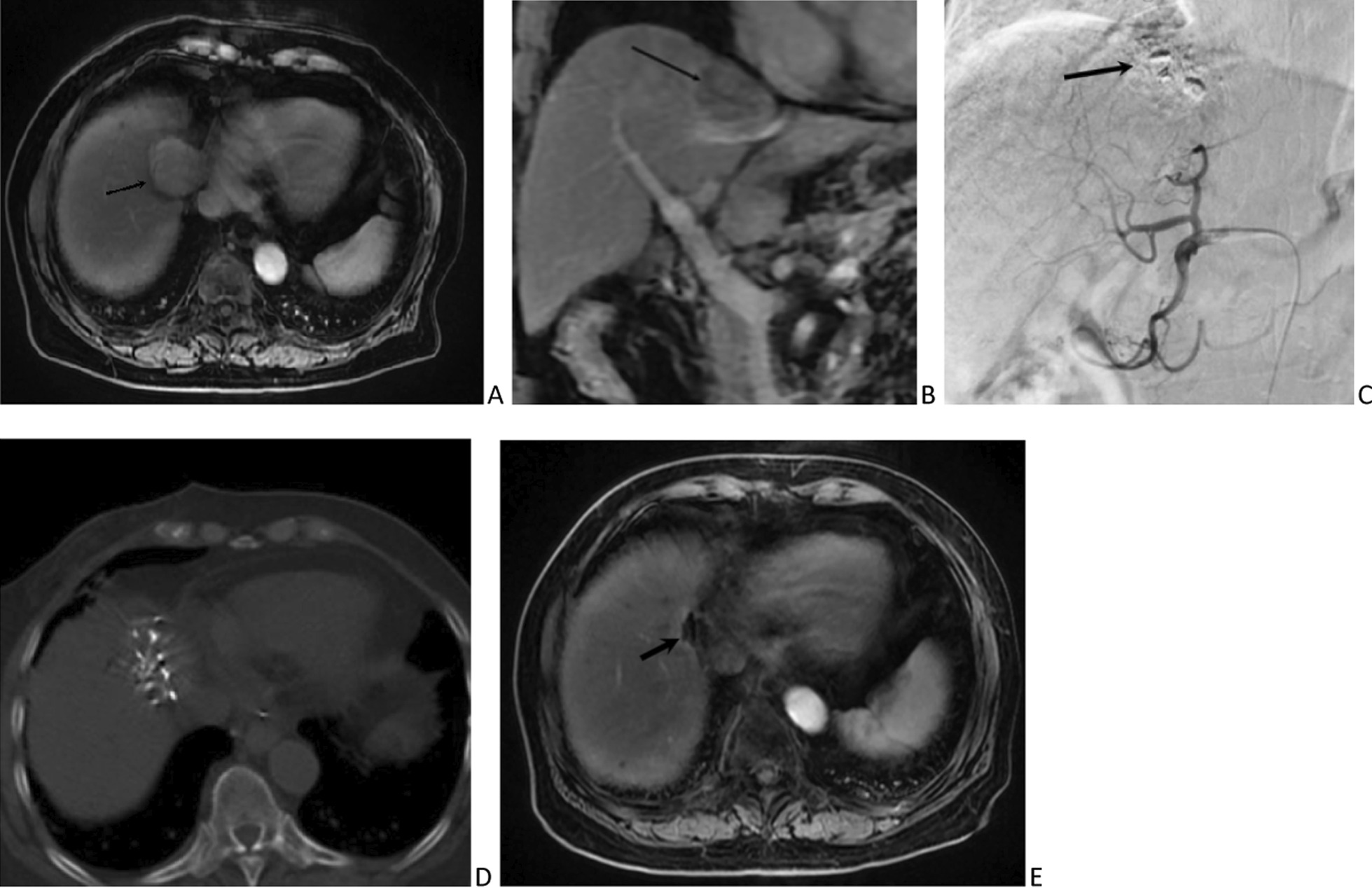
A 78-year-old female with subcapsular HCC underwent combined TACE and ISB treatment. Preoperative **a** axial and **b** coronal contrast-enhanced MRI showed the subcapsular HCC (arrows). **c** The procedure of TACE (arrow). **d** The ISB was performed by the CT guidance. **e** Postoperative contrast-enhanced MRI showed the shrinkage of the tumor with no intratumoral arterial enhancement (arrow). It could be considered as CR

In the combination group, the 32 individuals were inserted with 1560 I-125 seeds (mean 48.8 seeds/patient). Figure 4 showed the procedure of the combined treatments. After CT-guided ISB, only 2 (6.3%) patients experienced a self-limited pneumothorax.

### Treatment response

Table 2 demonstrates the treatment response results between the two groups. Combined treatment resulted in a considerably higher CR rate than the TACE alone did (56.3% vs. 18.8%, *P* = 0.002). Besides, the total response rate was notably greater in the combination treatment group compared with that in TACE alone group (90.7% vs. 59.4%, *P* = 0.004).

**Table 2:** Comparison of treatment response between 2 groups

	Combined treatment	TACE alone	*P* value
Complete response	18 (56.3%)	6 (18.8%)	0.002
Partial response	11 (34.4%)	13 (40.6%)	0.606
Stable disease	1 (3.1%)	10 (31.3%)	0.003
Progression disease	2 (6.2%)	3 (9.3%)	1.000
Total response (complete + partial response)	29 (90.7%)	19 (59.4%)	0.004

*TACE* transcatheter arterial chemoembolization

### Survival

**Fig. 5:**
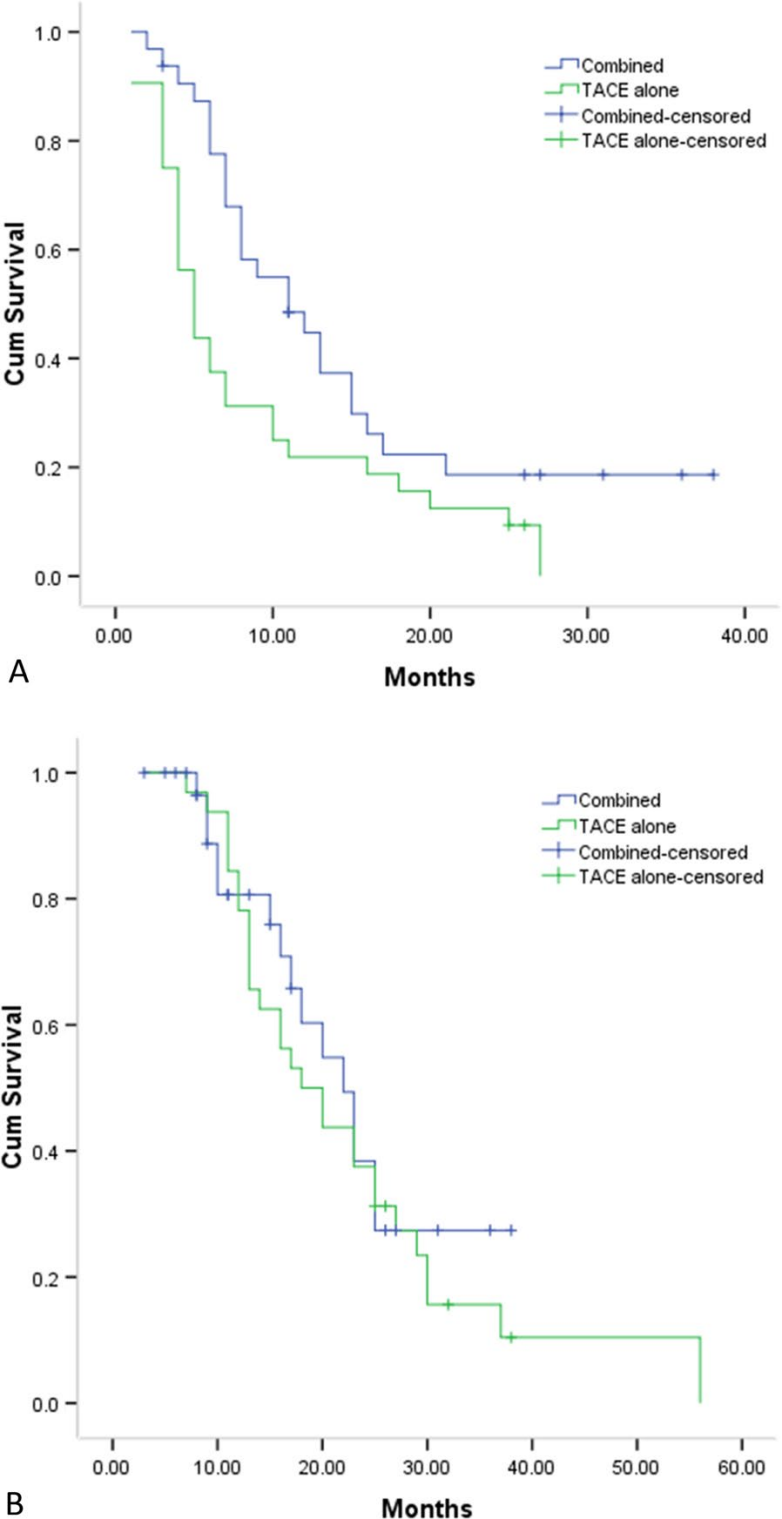
The comparison of PFS **a** and OS **b** period based on all patients

The median PFS was substantially longer in the combined treatment group in comparison to that in TACE alone group (11 months vs. 5 months, *P* = 0.016, Fig. 5a). The respective 1-, 2-, and 3-year PFS rates were 44.8%, 18.7%, and 18.7% in combined group and 21.9%, 12.5%, and 0.0% in TACE alone group, respectively.

Fifteen and 28 patients belonging respectively to the combined and TACE alone groups expired during follow-up. In all patients, the precise cause of death was tumor progression. The median OS was comparable between combined and TACE alone groups (22 months vs. 18 months, *P* = 0.529, Fig. 5b). The respective 1-, 2-, and 3-year OS rates were 80.6%, 38.4%, and 27.4% in combined group and 78.1%, 37.5%, and 15.6% in TACE alone group.

**Fig. 6:**
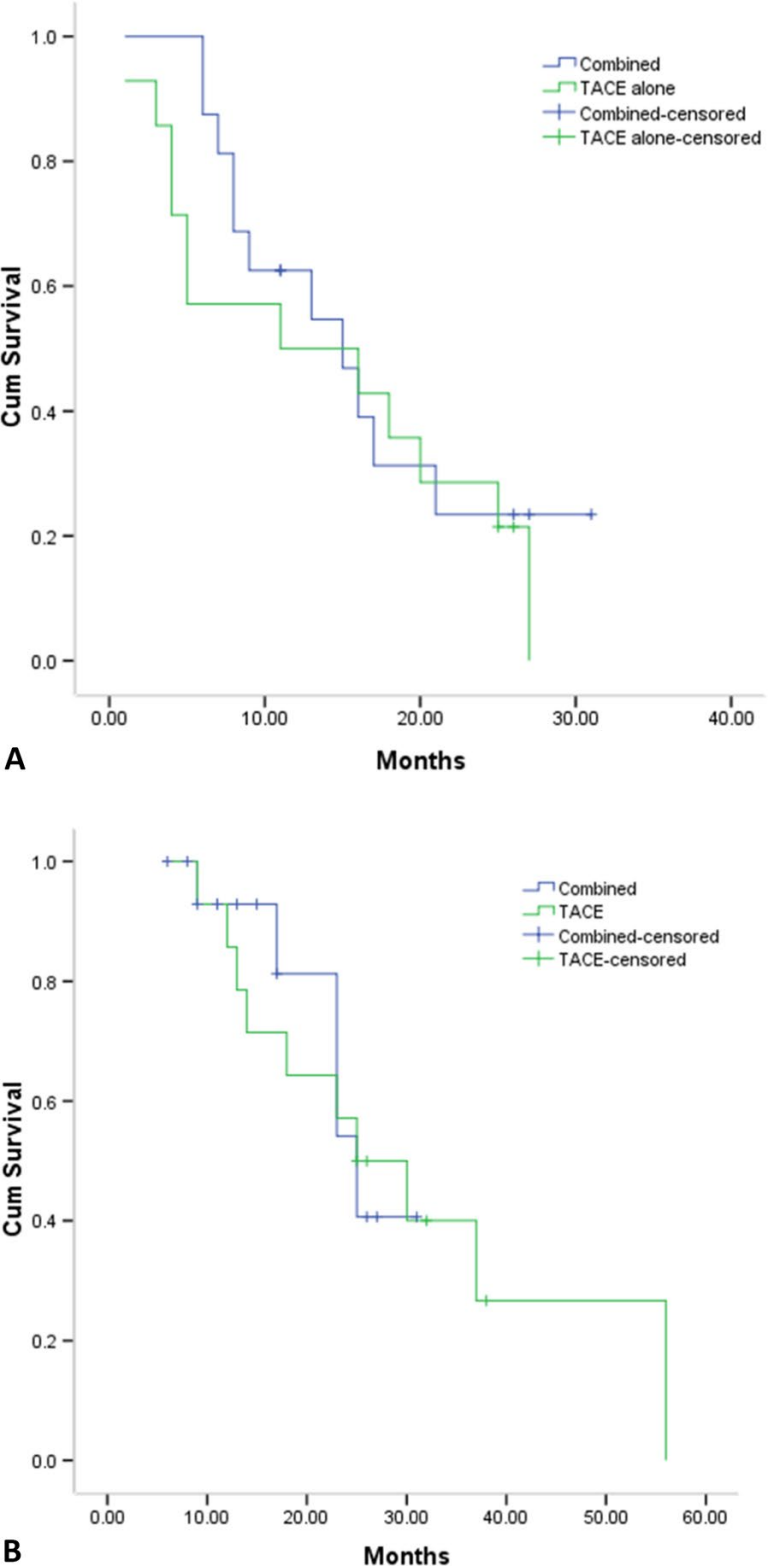
The comparison of PFS **a** and OS **b** period based on BCLC stage A patients

**Fig. 7:**
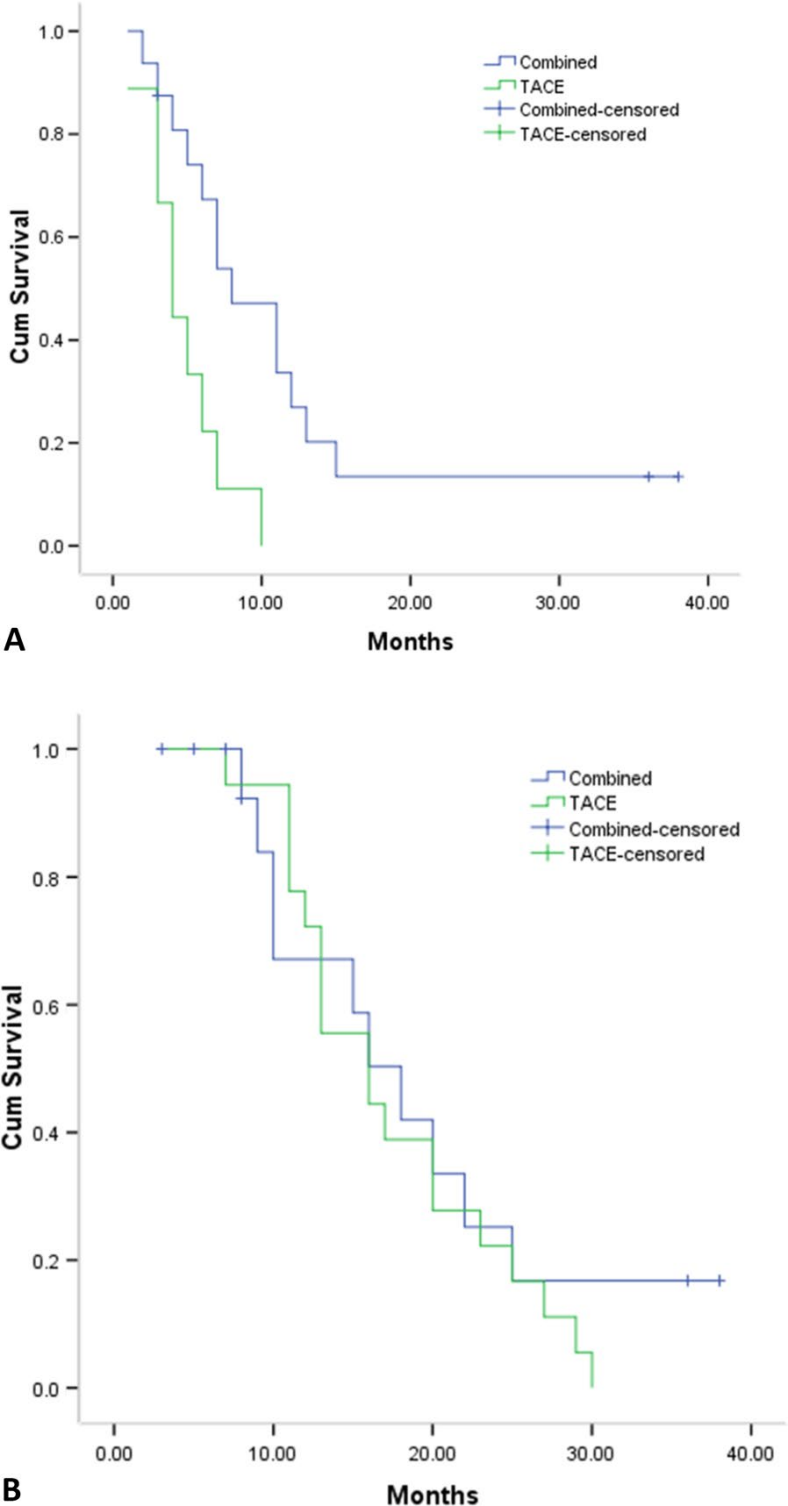
The comparison of PFS **a** and OS **b** period based on BCLC stage B/C patients

Based on the BCLC stage A patients, the median PFS (15 months vs. 11 months, *P* = 0.478, Fig. 6a) and OS (25 months vs. 25 months, *P* = 0.910, Fig. 6b) were both comparable between the two groups. Based on the BCLC stage B/C patients, the median PFS (8 months vs. 4 months, *P* = 0.001, Fig. 7a) was significantly longer in the combined group, while the OS (18 months vs. 16 months, *P* = 0.538, Fig. 7b) was comparable between the two groups.

Predictors of survival.

Based on univariate Cox-regression analysis, combined portal vein tumor thrombi (PVTT, *P* = 0.002), Barcelona clinic liver cancer (BCLC) stage B (*P* = 0.009), BCLC stage C (*P* < 0.001), and use of ISB (*P* = 0.022) were correlated with the PFS period. Based on multivariate Cox-regression analysis, BCLC stage B was the predictor of a shorter PFS period (*P* = 0.002, Table 3). The utilization of ISB was the predictor of a longer PFS period (*P* = 0.003, Table 3). Target treatment was not the predictor of longer PFS period (*P* = 0.223) on the basis of the univariate Cox-regression assessment.

**Table 3:** Predictors of progression-free survival

	Univariate analysis			Multivariate analysis		
	Hazard ratio	95% CI	*P* value	Hazard ratio	95% CI	*P* value
Tumor thrombi						
No	1			1		
Yes	3.837	1.658–8.878	0.002	4.480	0.922–21.759	0.063
BCLC stage						
A	1			1		
B	2.186	1.218–3.921	0.009	2.728	1.443–5.158	0.002
C	5.978	2.207–16.196	< 0.001	2.437	0.426 –13.958	0.317
Treatment protocols						
Combined	1			1		
TACE alone	1.884	1.095–3.240	0.022	2.392	1.297–4.413	0.003

*AFP* Alphafetoprotein, *BCLC* Barcelona clinic liver cancer, *TACE* transcatheter arterial chemoembolization

Regarding the univariate Cox-regression evaluation, tumor diameter (*P* = 0.008), combined PVTT (*P* = 0.002), BCLC stage B (*P* = 0.016), BCLC stage C (*P* < 0.001), and extra-hepatic metastasis (*P* = 0.005) were correlated with the OS period. Based on multivariate Cox-regression assessment, combined PVTT (*P* = 0.024) was the only predictor of a shorter OS period (Table 4). Target treatment was not the predictor of longer OS period (*P* = 0.378) according the univariate Cox-regression assessment.

**Table 4:** Predictors of overall survival

	Univariate analysis			Multivariate analysis		
	Hazard ratio	95% CI	*P* value	Hazard ratio	95% CI	*P* value
Diameter	1.182	1.045–1.336	0.008	1.108	0.963–1.275	0.152
PVTT						
No	1			1		
Yes	7.511	2.662–21.199	< 0.001	14.094	1.418–140.098	0.024
BCLC stage						
A	1			1		
B	2.281	1.168–4.454	0.016	1.928	0.950–3.910	0.069
C	7.841	2.590–23.739	< 0.001	0.367	0.025–5.350	0.386
Extra-hepatic metastasis						
No	1			1		
Yes	10.775	2.089–55.568	0.005	4.274	0.566–32.275	0.159

*BCLC* Barcelona clinic liver cancer, *PVTT* portal vein tumor thrombi, *TACE* transcatheter arterial chemoembolization

### Toxicity effects

The National Cancer Institute's Common Toxicity Criteria grading version 2.0 was used as a reference for toxic effects [17]. Fever, vomit, and myelosuppression were the most common side effects. There existed no substantial discrepancies in rates of fever (43.8% vs. 46.9%, *P* = 0.802), vomit (37.5% vs. 31.3%, *P* = 0.599), and myelosuppression (28.1% vs. 28.1%, *P* = 1.000) between 2 groups.

## Discussion

Treatment of subcapsular HCC is rather challenging due to its location [18–20]. Various segments including the caudate lobe and the posterosuperior segments are complicated to be resected laparoscopically [21]. Although TACE can treat HCC at any location via the hepatic artery approach, many supplementary treatments, which include percutaneous ablation and ISB, have been employed to prolong the OS and PFS following TACE [22, 23]. Percutaneous ablation has been documented to be effective and safe for treatment for subcapsular HCC [18–20]. Nevertheless, complications including right pleural effusion, transient lung change, right shoulder pain, diaphragmatic thickening, and subsegmental intrahepatic bile duct stricture have been frequently described [13].

The current work is based on an assessment of the safety and clinical efficacy of combined TACE and ISB for subcapsular HCC. First of all, only 2 patients (6.3%) experienced self-limited pneumothorax and this result suggests the safety of CT-guided ISB for subcapsular HCC. The complication rate in our study is comparable to other studies using percutaneous ablation for subcapsular HCC [18–20], where the mean complication rate was approximately 10.8%. However, to preserve the surrounding organ during percutaneous ablation for subcapsular HCC, the hydrodissection approach was commonly adopted [18]. ISB, unlike percutaneous ablation, does not necessitate this approach [13].

In this study, the CR rate was significantly greater in the combination group (*P* = 0.002), while the PR rates were comparable between the two groups (*P* = 0.606). Based on the result of PR rates, we found that TACE alone could effectively inhibit the tumor progression, while the CR rates indicated that ISB based on TACE can further kill the tumor cells. Furthermore, the total response rate of the combination group in this study (90.7%) was comparable to that (80.95%) in a previous study based on TACE and ISB used in combination for HCC [24].

PFS is a metric that measures the ability of long-term control. Our findings suggest that ISB could help TACE maintain long-term control. This could be because I-125 seeds emit gamma radiation, which can destroy cycle-sensitive cells, and low-dose radiation has an effect on tumor cell distribution, making HCC cells more sensitive to chemotherapy and boosting long-term efficacy. ISB, on the other hand, had no effect on PFS in individuals with BCLC stage A HCC. The possible reasons for this result are (a) the sample size is small; (b) TACE alone may also effectively control the smaller or early-stage HCC [25].

The 1-year and 3-year OS rates (80.6% and 27.4%) after combined treatment in our study are roughly comparable to those (87.9% and 46.7%) in a previous study regarding the use of TACE and ISB in combination for HCC [22]. Furthermore, these rates are also comparable to the study regarding TACE with microwave ablation (89.5% and 32.6%) for HCC [26]. Our comparative results, on the other hand, showed that ISB did not improve the OS period following TACE. This result could be ascribed to the limited sample size. Moreover, this result could mainly be explained by the fact that the TACE cycles were substantially greater in the TACE alone group (*P* = 0.001). Because the TACE alone group had a lower CR rate and higher tumor progression rate, additional TACE cycles were conducted timely to control the tumor progression. In other words, we can expect ISB to aid in the reduction of TACE use. Furthermore, when paired with PVTT, it was observed to be associated with a shorter OS time. PVTT is frequently associated with a bad prognosis as a result of tumor growth, as well as decreased portal circulation and elevated portal pressure [16].

Fever, vomit, and myelosuppression are all prominent adverse consequences of TACE. ISB did not increase the toxicity of TACE, as suggested by our findings. These results could be ascribed to the usage of TPS prior to ISB. The number and distribution of the I-125 seeds were designed by TPS and the radiation dose to the adjacent non-tumor tissues was controlled to a minimum [22].

At present, transarterial radio-embolization (TARE) using the Yttrium-90 (Y90) has been broadly employed for treating inoperable HCC and intra-hepatic cholangiocarcinoma (ICC) [27–29]. Based on the results of a recent meta-analysis, compared to TACE, TARE could provide the similar good outcomes with the significant lower adverse event rates for patients with ICC [29]. A randomized controlled trial showed that TARE could provide significant longer time-to-progression than TACE did (26 months vs. 6.8 months, *P* = 0.012) for patients with HCC [30]. However, the clinical effectiveness between ISB and TARE should be further confirmed.

The study has a few limitations. To begin, this is entirely a retrospective analysis which usually results in a high risk of selection, comparability, and exposure bias. Second, the rates of HBsAg (+) were not comparable between the two groups. Therefore, we performed the Cox-regression analyses and found that HBsAg (+) was not associated to the survival period. Despite the fact that this item was not linked to PFS or OS, this finding enhanced the likelihood of bias. Third, because the sample size of BCLC stage C patients was rather limited, we did not estimate the PFS and OS for these patients. Hence there is a definite need for more well-designed prospective investigations with relatively large sample sizes.

## Conclusion

Briefly, combination of TACE and ISB is a safe treatment method for subcapsular HCC. The clinical efficacy of combination treatment was significantly superior to TACE alone in the treatment of subcapsular HCC.

## Data Availability

The data that support the findings of this study are available from the corresponding author upon reasonable request.

## Abbreviations

BCLC: Barcelona clinic liver cancer
CT: Computed tomography
HCC: Hepatocellular carcinoma
ISB: I-125 seeds brachytherapy
MRI: Magnetic resonance imaging
OS: Overall survival
PFS: Progression-free survival
PVTT: Portal vein tumor thrombi
TACE: Transarterial chemoembolization
TARE: Transarterial radio-embolization
